# Affordable mobile microfluidic diagnostics: minimum requirements for smartphones and digital imaging for colorimetric and fluorometric anti-dengue and anti-SARS-CoV-2 antibody detection

**DOI:** 10.12688/wellcomeopenres.16628.1

**Published:** 2021-03-11

**Authors:** Sophie M. Jégouic, Ian M. Jones, Alexander D. Edwards

**Affiliations:** 1 Reading School of Pharmacy, University of Reading, Reading, RG6 1EE, UK; 2 School of Biological Sciences, University of Reading, Reading, UK

**Keywords:** smartphone, diagnostics, point-of-care, microfluidics, infection

## Abstract

**Background:** Miniaturised bioassays permit diagnostic testing near the patient, and the results can be recorded digitally using inexpensive cameras including smartphone and mobile phone cameras. Although digital cameras are now inexpensive and portable, the minimum performance required for microfluidic diagnostic bioassays has not been defined. We present a systematic comparison of a wide range of different digital cameras for capturing and measuring results of microfluidic bioassays and describe a framework to specify performance requirements to quantify immunoassays.

**Methods:** A set of 200 µm diameter microchannels was filled with a range of concentrations of dyes used in colorimetric and fluorometric enzyme immunoassays. These were imaged in parallel using cameras of varying cost and performance ranging from <£30 to >£500.

**Results:** Higher resolution imaging allowed larger numbers of microdevices to be resolved and analysed in a single image. In contrast, low quality cameras were still able to quantify results but for fewer samples. In some cases, an additional macro lens was added to focus closely. If image resolution was sufficient to identify individual microfluidic channels as separate lines, all cameras were able to quantify a similar range of concentrations of both colorimetric and fluorometric dyes. However, the mid-range cameras performed better, with the lowest cost cameras only allowing one or two samples to be quantified per image. Consistent with these findings, we demonstrate that quantitation (to determine endpoint titre) of antibodies against dengue and severe acute respiratory syndrome coronavirus 2 (SARS-CoV-2) viruses is possible using a wide range of digital imaging devices including the mid-range smartphone iPhone 6S and a budget Android smartphone costing <£50.

**Conclusions:** In conclusion, while more expensive and higher quality cameras allow larger numbers of devices to be simultaneously imaged, even the lowest resolution and cheapest cameras were sufficient to record and quantify immunoassay results.

## Introduction

The use of digital cameras in consumer devices, such as smartphones, to record the results of miniaturised bioassays, offers many potential advantages in clinical diagnostics. Smartphones can digitally record, interpret and quantify many measurements that have previously relied on a laboratory instrument to analyse, or needed a human operator to interpret lines or colour change by eye and then manually record results. A wide spectrum of devices and bioassays have been read using smartphones ^1^, including pH in clinical samples ^2^ immunoassays ^3, 4^, nucleic acid detection ^5, 6^, microbiology ^7, 8^, and paper devices including urine analysis dipsticks ^9^ or cholesterol test strips ^10^. Smartphones offer a combination of three features in an accessible package: high performance camera, on-board computing power, and networking. Firstly, optimised miniaturised optics combined with high-performance complementary metal oxide semiconductor (CMOS) image sensors deliver increasingly high-performance digital cameras. Secondly, constantly advancing processors deliver local fast and powerful computing capacity, plus onboard random-access memory (RAM) and secure digital (SD) card data storage allow image acquisition, storage, processing and analysis. Thirdly, mobile network bandwidth and expansion of wireless networking permits integration to connected health systems and – if handset computing power is insufficient- cloud-based analysis of results. The latter two features can also be used without the camera to power external sensor modules ^11^. Bioassays read by smartphone can aid diagnosis either as stand-alone point-of-care clinical measurement ^12^, or results can be combined with other clinical data where smartphones have been explored for providing clinical guidance in the field ^13, 14^.

The large consumer markets and massive sales volumes have driven up hardware performance and cut optoelectronic component price. However, the latest high-end products with highest hardware performance and most handset features comes at increased price, with cost of many high-end smartphone handsets exceeding £1000. Alternative, lower price and performance options have followed. Two classes of low-budget mobile phone handset have emerged. Firstly, in many regions of the world (e.g. India, many African states) mobile phones without the networking and microprocessor power of smartphones, termed “feature phones” have been the most widely owned handsets ^15^. These offer longer battery life and are better suited to users and regions where network capacity is limited and communication by phone/SMS remains the most important driver for mobile phone ownership. Secondly, budget smartphones have proliferated, especially those with android operating system, with reduced features (e.g. older operating systems) and less highly specified hardware (less memory, simpler screen, lower resolution camera, cheap casework). Touchscreen, fully networked android handsets are available under £50. Whilst many feature phones do have cameras, the limited screen size and memory ensures these are hard to use and they are typically equipped with very simple low-resolution optoelectronics.

Consumer products such as smartphones can be hard to adopt into diagnostics because their closed and rapidly changing software, and variable hardware specification, is not compatible with tightly regulated and standardised requirements for regulatory approved diagnostics. An alternative to using consumer products is to exploit the underlying hardware (e.g. optoelectronic components) to build bespoke, regulatory approved, camera-based readers/analysers. The CMOS sensors and lenses found within smartphones are increasingly available as camera modules. The most readily available are bundled with single-board computers such as the Raspberry Pi, or with microcontrollers. Industrial use of digital cameras for example in manufacturing for machine vision has likewise grown accelerated by the fall in cost of cameras and network/processing power ^16^. The cameras used in industrial applications have one major advantage over consumer products, which is greater robustness and manufacturing specifications for longer-term use coupled with more software control, compared to the short product life for consumer products where profit is driven by frequent replacements. Furthermore, smartphone camera software optimises images for consumer preference- not diagnostic accuracy. However, this comes at a cost, with machine vision cameras typically costing far more for equivalent sensors.

As many studies ^17– 19^ have reported proof-of-concept demonstrating that smartphone imaging can be used to read microfluidic assays a systematic comparison of different digital camera hardware is warranted to understand the key parameters for digital imaging of bioassays. We developed a simple and low-cost microfluidic platform that exploits the optical transparency of melt-extruded fluoropolymer microcapillary film (MCF). We previously showed both smartphone and consumer digital cameras, as well as the Raspberry Pi camera, can quantify immunoassay and analytical microbiology assays within affordable microfluidic devices made from MCF ^3, 20– 24^. The low cost of the devices and the optical transparency achieved through refractive index matching makes this simple platform ideally suited to systematic comparison of imaging performance.

Here, we compared the analytical performance of a range of digital cameras and tested if lower cost feature phones and budget smartphone handsets were capable of recording colorimetric and fluorometric dyes within microfluidic devices. Using MCF as an example of microfluidic bioassays, we systematically compared the optical sensitivity, resolution and therefore analytical performance of these low-cost units against higher-specified smartphones and higher performance digital cameras. We also compared these consumer products with an industrial camera module developed for machine vision applications and a Raspberry Pi camera. Finally, we assessed if these digital cameras could measure simulated antibody responses against two viral infections of global health significance- dengue fever and coronavirus disease 2019 (COVID-19).

## Methods

### Experimental approach

**Figure 1.**
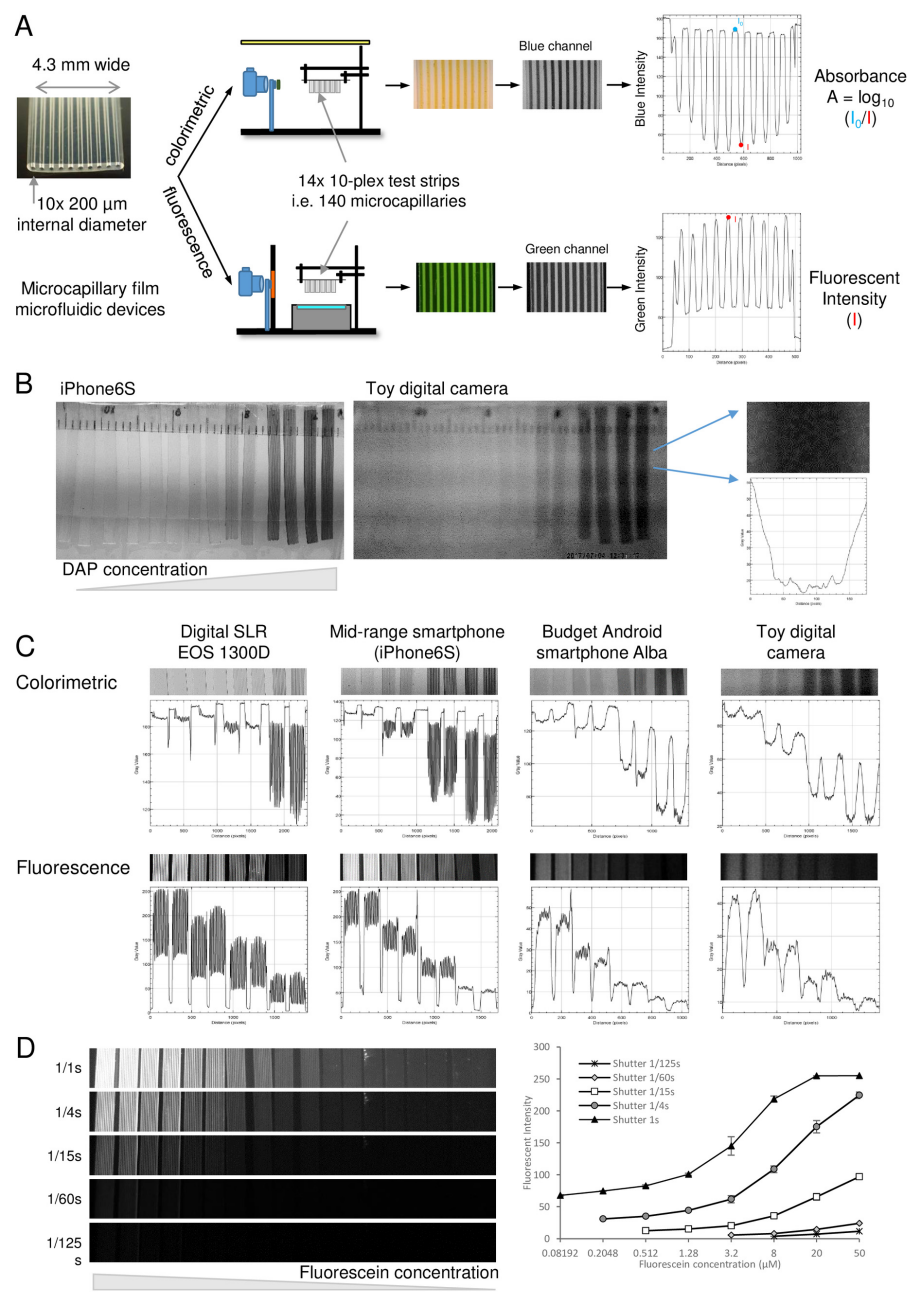
Experimental setup to systematically compare the digital image quantitation of microfluidic bioassays by different cameras and smartphones. **A**) Microcapillary film strips were used as examples of microfluidic devices capable of performing immunoassays. Sets of colorimetric or fluorescent dye solutions were loaded in a panel of strips and imaged either on a white light background for colorimetric (absorbance) measurement, or with blue light emitting diode (LED) excitation imaged through an amber emission filter for fluorescent. The absorbance or fluorescence intensity for each capillary was analysed for the appropriate red-green-blue (RGB) colour channel using ImageJ as indicated. **B**) Comparison of image quality for mid-range smartphone vs the cheapest camera, illustrating how lower quality images cannot resolve individual capillaries but variation in intensity is still clear. **C**) Illustration of systematic comparison of cameras for imaging microfluidic bioassays. **D**) effect of camera settings on fluorescence detection, showing that varying shutter speed can dramatically alter dynamic range of fluorophore measurement. The same set of serial fluorescein dilutions were imaged with the indicated range of shutter speeds. Mean fluorescence intensity for 20 replicate capillaries in duplicate microcapillary film (MCF) test strips were plotted, with error bars indicating standard deviation.

We established a simple imaging rig to systematically determine the performance of a range of digital cameras for imaging colorimetric and fluorometric assays within microfluidic devices ( Figure 1A). To allow us to control the microfluidic device characteristics and directly compare camera performances, we used colorimetric and fluorescent dye solutions- rather than full immunoassays where substrate conversion is dynamic and target dye concentrations are uncontrolled. Di-amino phenazine (DAP) is the yellow dye product produced by the horseradish peroxidase enzyme, commonly used in colorimetric immunoassays, acting upon the o-Phenylenediamine dihydrochloride substrate (OPD) substrate often used in colorimetric immunoassays. Fluorescein is used both directly for fluorescent detection and also as the product of alkaline phosphatase conversion of the substrate fluorescein di-phosphate (FDP) and is spectrally similar to other alkaline phosphatase substrates such as Attophos™. Following this systematic comparison using dyes, selected cameras were used to image full immunoassays that simulated the measurement of antibodies against important viral antigens, to confirm the findings could be applied to clinically relevant diagnostics bioassays. For all the cameras but the G:Box, the images were taken at about 10cm from the subject and using a digital setting of 1 (equivalent to no zoom). The distance of the camera to the sample was recorded and this working distance shown for different cameras below alongside example images. Automatic settings were used for the Raspberry Pi 3B+, the toy camera, the Alba Phone and the iPhone 6S. Manual settings were used to take images with the Powershot S120, with a fixed ISO of 3200 and variable F-Stop and exposure.

***Target microfluidic devices for fluorometric and colorimetric signal detection.*** Whilst the characteristics of the microchannels within MCF are typical of many microfluidic devices, with assay channels of 200 µm internal diameter, they have the advantage of low cost allowing large numbers of devices to be filled with differing dye concentrations and simultaneously imaged. They have some unique features including a cylindrical cross-section that results in an elliptical intensity plot when projected onto a flat image, and refractive index matching between fluoropolymer device and water, avoiding distortion at the interface between aqueous sample and microdevice. Previous studies have shown these permit a wide range of bioassays to be conducted including immunoassays and analytical microbiology.

### Materials and reagents

The MCF was manufactured by Lamina Dielectrics Ltd (Billingshurst, West Sussex, UK) and consisted of an array of 10 micro-capillaries produced from Teflon®-FEP FEP (fluorinated ethylene propylene) (Dow, USA) using a continuous melt-extrusion process ^22, 23^. The MCF had a width of 4.3mm and the mean internal diameter of the microcapillaries was 206µm. Fluorescein NIST-Traceable Standard (ThermoFisher Scientific, UK) was used to assess the cameras/phones performance to detect fluorescence and was used as a reference for the immunoassays. A stock solution of 100mM 2,3-Diaminophenazine (DAP) (Sigma, UK) was prepared in DMSO. Dilutions of DAP were prepared in SIGMA-FAST OPD Buffer (Sigma, UK). DAP was used to assess the cameras/phones performance to detect colorimetric signals. A 2 mg/mL solution of high molecular weight polyvinyl alcohol (PVOH, Sigma, UK) in PBS was used to coat the inner surface of the micro-capillaries to make them hydrophilic ^22, 23^. Human negative control serum was bought from Sigma. The blocking buffer consisted of Superblock (ThermoFisher Scientific, UK) supplemented with 10% foetal bovine serum (Sigma, UK) and 5% goat serum (Sigma, UK). Positive control antiviral antibodies were recombinant human IgG and IgM anti-Flavivirus group antigen [D1-4G2-4-15 (4G2)] and human IgM Anti-COVID-19 & SARS-CoV S glycoprotein [CR3022], obtained from Absolute Antibody (Oxford, UK). Anti-human IgG AP and anti-human IgM Ap secondary antibodies were purchased from ThermoFisher Scientific (UK). For imaging both dengue virus (DENV) and SARS-CoV-2 immunoassay strips, AttoPhos® AP Fluorescent Substrate System (Promega, UK) was added as the final substrate. Baculovirus expression of viral proteins used *Spodoptera frugiperda* ( *Sf9*) and *T.nao38* cells which were maintained in EX-CELL 420 medium (Sigma, UK) supplemented with 2% fetal bovine serum (Sigma, UK), at 27°C with shaking. Virus growth used exclusively *Sf9* cells while protein expression used *T.nao38* cells.

### Digital cameras, mobile phones, and imaging devices

For the detection of fluorescent and colorimetric signals, we used a wide range of cameras and phones, and professional imaging systems ( Table 1). We used a DSLR camera EOS 1300D with a Canon EF-S 60mm f/2.8 Macro USM Lens (Canon), a compact camera Powershot S120 (Canon), a compact waterproof and shock-resistant WG4 camera (Ricoh) and a toy camera (Sakar). We used smartphones including an iPhones 6S and 4S (Apple) and Alba SIM Free 5'' Android and mobile phones such as CAT B30 Phone (Caterpillar) and Alcatel 2008G phone (Alcatel) purchased from Argos (UK). The industrial machine vision camera USB 3 uEye® XC with a Macro Lens (AE00126; IDS Imaging Development Systems, Obersulm, Germany), a Raspberry Pi camera module v2 powered by a Raspberry Pi 3 B+ computer (Raspberry Pi Foundation, UK) and the laboratory imaging systems G:BOX (Syngene, UK) and Typhoon (Amersham, UK) were also used to detect fluorescent and colorimetric signals. Images were taken at a resolution of 3280x2464 pixels with the Raspberry Pi camera.

**Table 1.** Cost and properties of different classes of digital cameras.

Market	Product	Type	Class	Price Range	Brand/Camera	Price when purchased	Sensor Size	Image dimensions (pixels)	Stated Sensor Resolution (Megapixels)	Aperture	Focal length (35mm equivalent)
Consumer	Mobile phone camera	Smartphone	Flagship	> £700	Samsung/Galaxy S9+ *		1/2.55"		12	f/1.5-2.4	26 mm
			Mid-range	£400 - £700	Apple/iPhone 6S *		1/3"		12	f/2.2	29 mm
			Budget	£50 - £400	Apple/iPhone 4S *		1/3.2"		8	f/2.4	33 mm
					Alba/SIM Free 5'' Android ^TM^ *	£50	unknown	1920 x 2560	5	f/2.8	unknown
		Phone			Caterpillar/CAT B30 Phone	£80	unknown	1200 x 1600	2	unknown	unknown
			Feature phone	< £50	Alcatel/2008G	£35	unknown		2	unknown	unknown
	Digital Camera	DSLR	Flagship	£250 - £1000	Canon/EOS 1300D + Canon EF-S 60mm f/2.8 Macro USM Lens	£570 including macro lens	22.3 x 14.9 mm		18	f/2.8-32	96 mm
		Compact	Mid-range	£100 - £250	Canon/Powershot S120	£250	1/1.7" (~ 7.53 x 5.64 mm)		12.1	f/1.8-5.7	24 - 120 mm 5.2 - 26 mm
					Ricoh/Compact WG4	£280	1/2.3" (~ 6.16 x 4.62 mm)		16	f/2-4.9	25 - 100 mm
		Kids camera	Budget/Toy	< £50	Toy camera (CA2-10027 Frozen)	£25	unknown	3648 x 2736	10.1	f/2.8	4.1mm
	Camera powered by single-board computer		Budget	< £50	Raspberry Pi Ltd/v2 camera Raspberry Pi 3 B+	£33 for computer plus £22 for camera	1/4"		8	f/2.0	(3.60 mm – not 35mm equivalent)
Industrial	Machine vision	Webcam		≥ £150	IDS/USB 3 uEye® XC + Macro Lens (AE00126)	£330 plus £30 for lens	1/2.45	4192 x 3104	13	f/2.8mm	5.3 mm
Laboratory	Gel Doc system			> £5000	Syngene/G:Box		unknown	1391 x 1039	4	unknown	unknown
					Amersham/Typhoon		unknown		N/A	N/A	N/A

To improve the image quality and resolution of some imaging systems by allowing closer focussing on the microfluidic channels, three type of lenses were used: a simple injection-moulded plastic magnifying lens, a smartphone clip-on macro lens (Amazon, UK) and an industrial machine vision macro lens (IDS). These were held directly onto the front of the digital camera lens during imaging.

### Evaluation of digital imaging performance for colorimetric and fluorescent microfluidic assay measurement

MCF strips were prepared by firstly giving an internal hydrophilic coating with PVOH incubated at room temperature (RT) overnight, followed by washing and cutting into individual 75mm long test strips ^22^. For the colorimetric signal detection, a 4mM solution of DAP and 5-fold dilutions in SIGMA-FAST OPD Buffer were added to the MCF strips, in duplicate, using a 10ml syringe. The images were taken under a white light. The absorbance was calculated from the drop in blue light intensities measured using ImageJ software ^25^ to plot intensity across the microdevice, with the lowest point being taken as maximal absorbance value ( Figure 1A) ^3, 20, 21^. For the fluorescent signal detection, 50 uM Fluorescein and 5-fold dilutions in H2O were added to the MCF strips, in duplicate, by aspiration with a 10ml syringe. The images were taken using a light-emitting diode (LED) Transilluminator (IO Rodeo, USA) to provide blue light excitation in a dark room with the amber emission filter held between the camera and the test strips. The peak fluorescence intensity was determined for each microcapillary in the green image channel using ImageJ software ( Figure 1A).

### Image data publication

All image files are published in the associated dataset for this paper ^26^, and can be accessed to assess the relative image quality of different camera types. The image examples used in the figures are constructed from cropped images of individual MCF test strips as outlined in the examples shown in Figure 1, but all original digital images recorded by the cameras are available as underlying data ^26^. A CSV data sheet summarises the image file names in this data set of over 170 original image files, and this lists the conditions and camera in each image file.

### Baculovirus expression and purification of DENV2-E and SARS-CoV-2-S1

The sequence of DENV2-E containing domains I and II (EI/II) (nt 1 to 891) (accession number NC_001474) was codon optimised for *Spodoptera frugiperda* cells and the honeybee melittin signal peptide was added upstream of the sequence. The sequence was flanked by 18bps at the 5’ and 3’ ends homologous to the intended expression vector, pTriEx1.1 before being ordered (IDT Europe, Belgium). The 3’ flanking nucleotides were also designed to fuse the EI/II ORF in frame to the vector’s 6xHis tag encoding sequence. The gene and the vector were assembled by recombination using the In-Fusion HD Cloning kit (Takara, USA). The assembly reaction was then used to transform NovaBlue Singles Competent Cells (EMD Millipore, UK). The sequence of SARS-CoV-2 S1 was obtained from the cloned full-length S sequence and was cloned into the expression vector pTriEx1.1 (EMD Millipore, UK) and characterised as described previously ^27^.

*Sf9* cells were transfected with the baculovirus expression vector FlashBAC Gold (Oxford Expression Technologies, UK) and with either DENV2-EI/II or SARS-CoV2-S1 constructs to produce recombinant baculovirusesr ^28^. Large-scale protein expression was performed by infecting 1L of *T.nao38* cells with a high titre stock of the recombinant baculovirus and incubated for 3-5 days at 27°C. After incubation the supernatant containing the secreted protein was harvested, clarified by centrifugation at 4,300xg for 20min and filtered through a 0.45um filter. The clear supernatant was supplemented with 0.5nM nickel sulphate before being loaded onto the Bio-Scale Mini Profinity IMAC Cartridge (Bio-Rad, UK). The elution was carried out at a flow rate of 2.5 ml/min with a gradient elution of 0.05–0.5M imidazole or 0.05-0.25M imidazole over 60 min for DENV2-EI/II or SARS-CoV-2-S1 respectively.

### Indirect ELISA for the detection of IgG and IgM

MCF strips were coated with 5 ug/ml of DENV2-EI/II or with 15 ug/ml SARS-CoV2-S1 and incubated at RT for minimum 1h. The MCF strips were then coated with 0.1mg/ml PVOH for 3h at RT before adding blocking buffer. The MCF strips were stored overnight at 4°C in blocking buffer. For the DENV2-E assays, high (100 µg/ml) and low (5 µg/ml) amount of monoclonal IgG or IgM anti-Flavivirus was spiked into human serum and compared with human serum with no added anti-Flavivirus antibody. For the SARS-CoV-2-S1, a high (50 µg/ml) and low (5 µg/ml) amount of monoclonal IgM anti-COVID-19 was spiked into human serum and compared with human serum with no added anti-COVID-19 antibody. In both cases, seroreactivity against the antigen was measured using a conventional endpoint titre protocol, whereby the serum samples were serially diluted in blocking buffer. The serum samples were incubated for 10–20 min, followed by two washed with PBS/T. Then, the secondary antibody anti-human IgG or IgM conjugated with alkaline phosphatase was added to the strips at a dilution of 1 µg/ml in blocking buffer. The secondary antibody was incubated for 10–20 min before being washed three times with PBS/T. Finally, AttoPhos® AP Fluorescent Substrate System (Promega) was added and the fluorescent signal was captured using various imaging systems and measured using ImageJ software. For every assay, a reference MCF strip containing 2uM of fluorescein was added and was used to normalise the fluorescent signals from the assays. The relative fluorescent intensity was determined by dividing the fluorescent intensity of the assay by the fluorescent intensity of the reference.

## Results and discussion

### A wide range of digital cameras are capable of recording and quantifying colorimetric and fluorometric microfluidic test results

We established a simple testing rig to systematically compare digital images of microfluidic devices with a wide range of different cameras of different costs and formats ( Figure 1A). Overall, we found that all digital cameras tested – even the lowest quality and cost – were capable of recording and distinguishing signal intensity of microfluidic test results to some extent. However, the optics and optoelectronics affected sharpness of image and sensitivity of detection for both fluorescent and coloured dyes. For example, the iPhone 6S gave sharp images with high enough resolution to clearly distinguish all capillaries, in contrast to the cheapest toy camera where all capillaries were blurred together ( Figure 1B). This indistinct image is likely a consequence of both poor optics unable to resolve the individual capillaries, and limitations of the image sensor showing noise and with a pixel size too large to distinguish capillaries. In spite of this poor image, a clear concentration-intensity relationship was still very clearly measurable for cameras across the range of cost and quality, from the digital SLR with largest image sensor and highest quality optics, through both mid-range and budget smartphones, to the cheapest toy camera ( Figure 1C). A concentration-intensity relationship could be clearly quantified for both colorimetric and fluorescent dyes. This indicates that the most important requirement for digital quantitation may be obtaining an image of sufficient quality to resolve the microchannels, but that even the simplest optical sensors can quantify intensity levels.

Whilst the imaging setup was standardised as far as possible, it was clear that camera settings significantly affect signal and made a big impact on both analytical sensitivity and dynamic range of detection. It was only possible to control camera settings such as exposure, aperture and focus for a subset of cameras. By increasing the exposure time (but with fixed aperture and ISO sensitivity), the analytical sensitivity of fluorescence detection increased significantly, with lower concentrations of fluorescein becoming detectable, alongside an increase in background ( Figure 1D). At the longest exposure, the highest concentrations of fluorescein became saturated, with the 8-bit image intensity scale providing a limit to the measurable dynamic range. For those cameras with automatically controlled image settings, we could still compare intensity between devices within the same image, but if different devices were imaged independently, a reference sample would become essential to normalise sample intensity between images taken at different times ^3^.

### Quantitative comparison of camera performance for digital imaging of colorimetric microfluidic tests

**Figure 2.**
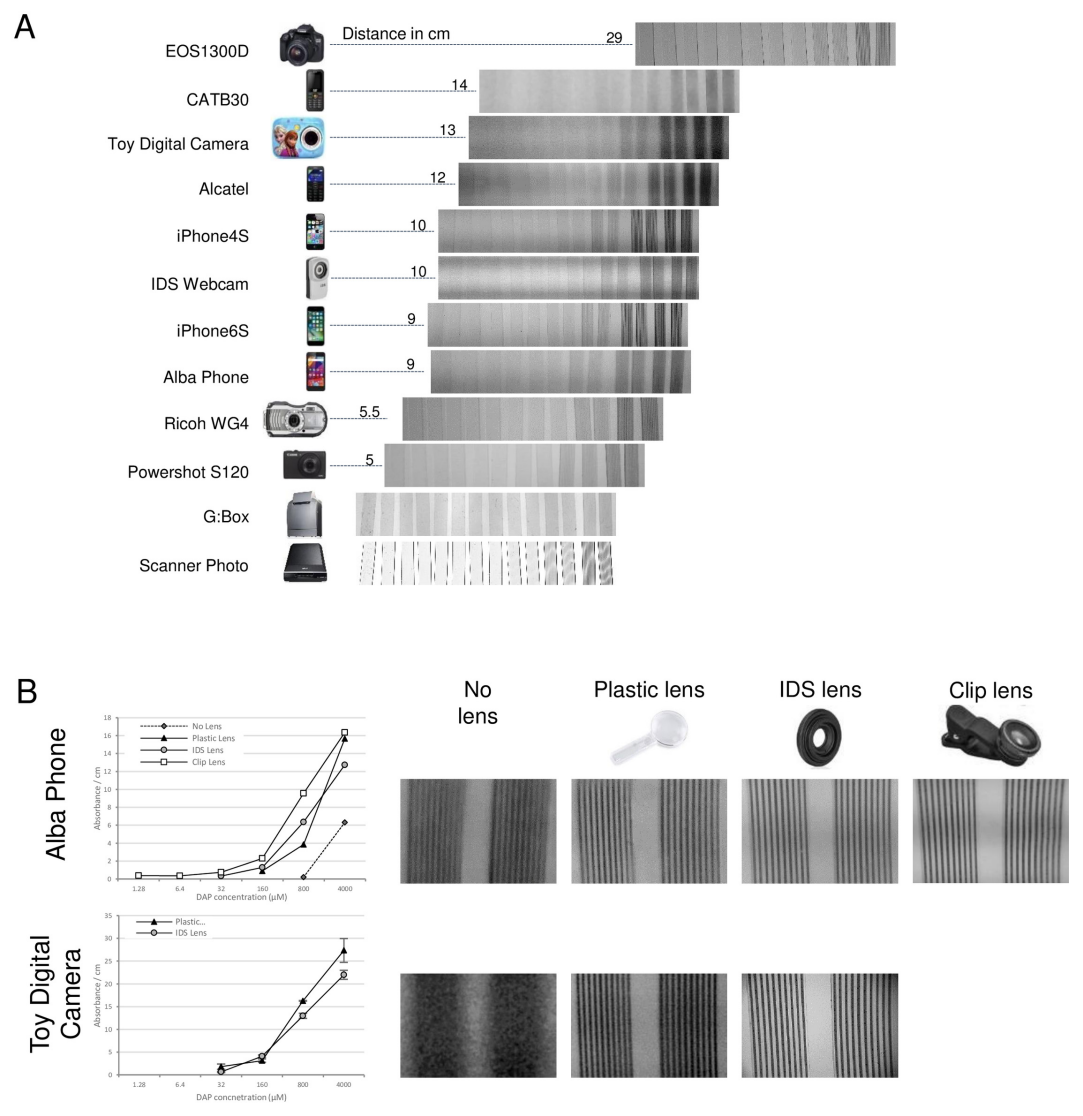
Example images of colorimetric microfluidic assays taken with range of digital cameras and phones. A series of five 5-fold concentrations of diaminophenazine dye (DAP) dye was made, representing the colorimetric product of horseradish peroxidase enzyme substrate conversion used in conventional enzyme immunoassays. These simulated immunoassay samples, plus a negative control, were loaded into pairs microcapillary film test strips. The full set of 12 samples were imaged using a range of different digital cameras, plus a laboratory gel scanner and consumer A4 flatbed scanner. **A**) example images illustrating the image quality are shown, with the blue channel where maximal absorbance of DAP presented. The distance of the camera to the sample to achieve an appropriate field of view is indicated in cm. **B**) Two examples of lower quality cameras, where individual capillaries could not be resolved when imaging the full set of 12 samples, so an additional macro lens was added allowing closer focus that permitted dye concentration to be quantified.

For each camera tested representative images of colorimetric dye filled microcapillary devices on a white light background are shown, listed in order of the working distance required to image the full set of microcapillary devices ( Figure 2A). The working distance is also indicated in the figure, and relates to the field of view for each camera setup. When imaging this full set of samples, all cameras were clearly able to quantify the variation in signal intensity between strips to some degree, but individual capillaries could only be resolved with a subset of cameras. The iPhone 6s, the DSLR Camera EOS1300D and the Powershot S120 were the best cameras to image a full of set of samples at high resolution. In contrast, the low cost phones (CATB30, Alcatel and Alba phones) and the toy camera provided poor resolution images which could not be analysed.

For the lower performance cameras with images where the resolution was too low to resolve individual capillaries multiple devices, we added an additional lens to permit closer focus. With the budget smartphone (Alba Phone) the capillaries were clearly distinguishable at higher dye concentrations but not as sharp as the higher performance cameras, and with the cheapest toy camera it was impossible to resolve any different capillaries in spite of the clear difference in intensity visible between strips of different concentration. Nevertheless, simply by adding additional plastic convex lens – sold as “macro lens” for addition to small cameras and as clip-on lenses for smartphones – it was possible to clearly resolve the individual capillaries when only a few strips were imaged ( Figure 2B). Three additional lenses were compared: the cheapest was an injection moulded magnifying lens that allowed focus on 6 test strips (around 35mm field of view); whereas both the professional macro lens for machine vision imaging and the consumer smartphone clip-on macro lens allowed focus on 3 test strips (around 15–20mm field of view). Although the clip-on lens could not be used with the toy camera, the benefits of macro lens addition for closer focus was clear for both the budget smartphone and toy camera, and it was possible to quantify absorbance within 200 µm diameter microfluidic channels with both of these very low cost cameras- in spite of their low quality image sensor and optics. Whilst the addition of macro lens made it possible to resolve individual microcapillaries and quantify colorimetric assays ( Figure 2B), there was a significant trade-off as closer focusing gave a smaller field of view and significantly reduced the number of microfluidic devices that could be simultaneously recorded.

In conclusion, the camera type significantly influenced the number of microfluidic devices that could be recorded simultaneously, with the lower the quality of optics and digital sensor, the fewer the microdevice measurements that can be captured in one single image. However, as long as the camera was able to resolve individual microchannels, dye quantitation was possible. All original image files are shared through the experimental dataset accompanying this paper.

**Figure 3.**
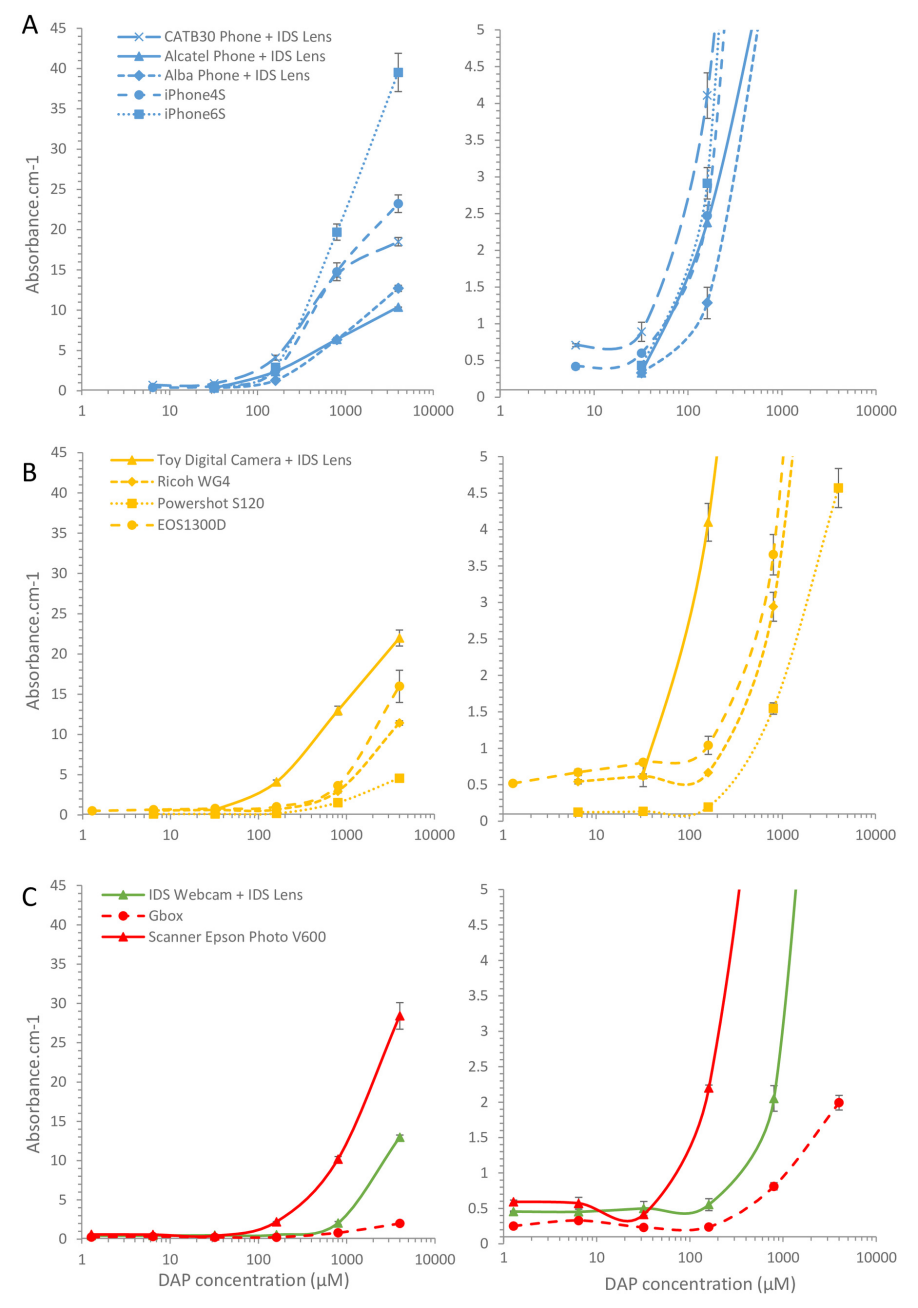
Quantitation of colorimetric bioassay signal by different digital cameras. Absorbance was calculated for the images in Figure 2 from the reduction in blue channel intensity for each individual capillary. The mean absorbance for 10 individual capillaries was then plotted against concentration, to illustrate the relative analytical performance for microfluidic colorimetric bioassay measurement by a range of digital cameras. **A**) Comparison of feature phones vs smartphones; where indicated a macro lens was used to be able to resolve individual capillaries. **B**) Comparison of consumer digital cameras. C) Comparison of industrial camera vs laboratory scanners vs consumer flatbed scanner. Error bars indicate the standard deviation of 10 capillaries. Lower absorbance values are plotted (Right) with smaller y-axis range to permit comparison of detection of limiting concentrations of fluorophore.

***Performance differences and quantitation.*** Having established that multiple microcapillary devices could be imaged, with some resolution limitations for the lower quality cameras, colorimetric absorbance was quantified and plotted against concentration to determine the relative analytical performance of different cameras ( Figure 3). With the lower resolution images, every effort was made to record the intensity for each capillary where the test strip was clearly visible. However, in many strips capillaries with lower concentrations could not be distinguished and no intensity could be measured, and so data points were only recorded when the intensity value for individual capillaries could be clearly identified after plotting intensity profiles on ImageJ. Comparing camera phones and smartphones showed that the whole range of phone cameras tested were capable of recording and quantifying colorimetric dye in microfluidic devices ( Figure 3A). However, the entry-level smartphone plus two feature phones – mobile phones with simple cameras but without touchscreens and with simple button interface – required the macro lens to resolve individual capillaries and permit quantitation. This reduced the number of test strips that could be imaged simultaneously. With both entry-level and mid-range smartphones 14 devices each containing 10 microcapillaries were imaged (i.e. 140 data points), in contrast after adding the macro lens to the three lower-quality camera phones for closer focus reduced this to recording 4 or fewer devices measuring only 40 capillaries i.e. 3.5x less data per image.

Surprisingly, the better optics and larger sensor in the three digital cameras did not appear to improve colorimetric quantitation, and both compact digital cameras (Powershot S120 and Ricoh WG4) and the DSLR showed very limited difference in absorbance between the 140 µM and 32 µM concentrations, whereas all phone cameras showed a large absorbance change over this range. When equipped with a macro lens to focus closely, the toy camera also showed a clear difference in absorbance between these concentrations ( Figure 3A, B). The largest sensor and best optics found in the DSLR with macro lens did allow capillaries to be clearly identified with dye concentration as low as 1.3 µM and there was a clear increase in absorbance across the 1.3 µM to 140 µM range with this camera, but given the absorbance only increased 2-fold over this >100-fold increase in concentration, the gradient is barely steep enough for accurate quantitation. Thus, although colorimetric detection is clearly feasible with a wide range of digital cameras, with high resolution optics and larger sensors permitting capture of many devices, or lower quality digital imaging able to capture fewer devices, there may be limits to both analytical sensitivity and to measurable dynamic range. However, the dynamic range can be increased by varying camera settings such as exposure times and sensor sensitivity ( Figure 1D).

Finally, an industrial machine vision camera, a flatbed scanner, and a laboratory gel scanner were compared, none of which offered improved quantitation over the phone cameras or digital cameras ( Figure 3C). The industrial machine vision camera required the macro lens for closer focus and was only able to capture 4 devices per image, in contrast the flatbed scanner was capable of capturing very large numbers of devices, with at least 150 devices (each with 10 capillaries, i.e. 1500 microchannels) fitting on a single scan. It is possible with further optimisation of imaging or scanning conditions, these digital imaging devices would meet the performance of the digital cameras, however this demonstrates that consumer digital cameras are more than capable of recording quantitative bioassay readouts alongside industrial/laboratory devices.

### Comparison of camera performance for digital imaging of fluorometric microfluidic tests

**Figure 4.**
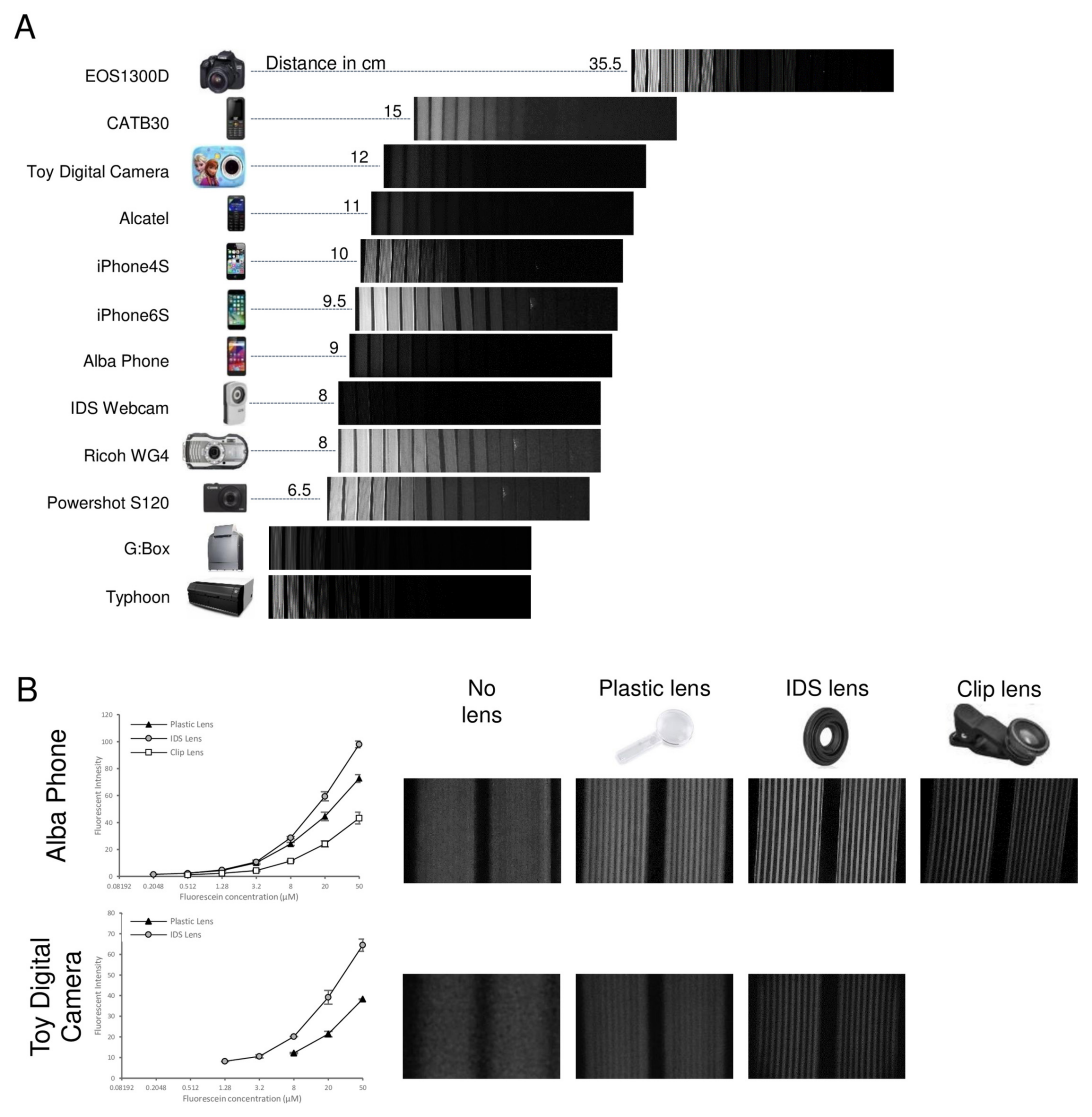
Example images of fluorescent microfluidic readout taken with a range of digital cameras and phones. A series of 5-fold dilutions of fluorescein were made to represent a range of results of fluorescent bioassays. A panel of microcapillary film strips filled with these fluorescein samples were imaged in parallel using the indicated range of cameras and lab scanners. **A**) example images for these cameras, with the distance from camera to sample to image the full sample set indicated in cm. **B**) For two cameras unable to resolve individual microcapillaries when imaging the full set of samples, macro lenses were added to allow closer focusing, with example images illustrating the improved resolution of macro images, and mean fluorescence of 10 replicate capillaries plotted to show how quantification of fluorescein remains possible even with these lowest performance cameras.

Fluorescent readouts can offer higher analytical sensitivity than colorimetric detection, although sensitivity of fluorescent detection depends significantly on excitation intensity and wavelength, quality of emission filters, in addition to the optical detector used. We found that even with the very simple and low-cost fluorescence imaging setup comprising an open source blue LED array transillumination device plus a simple amber acrylic emission screen, significantly lower concentrations of fluorescein were measurable – with some cameras clearly detecting 0.5 µM and lower – than with the colorimetric dye where the lowest measurable concentration was 10 µM or higher ( Figure 1D, Figure 4).

As with the colorimetric measurements, images with a wide range of cameras are compared in Figure 4, with the working distance needed to capture the full set of devices indicated. Although all cameras clearly recorded the changes in concentration between test strips, for many images the individual capillaries could not be resolved. As with colorimetric measurements, the macro lens allowed closer focussing and thereby facilitated quantitation of fluorescence within individual capillaries for the lower quality cameras, with even the cheapest toy camera capable of recording individual capillary intensity. All original image files are shared through the experimental dataset accompanying this paper ^26^.

**Figure 5.**
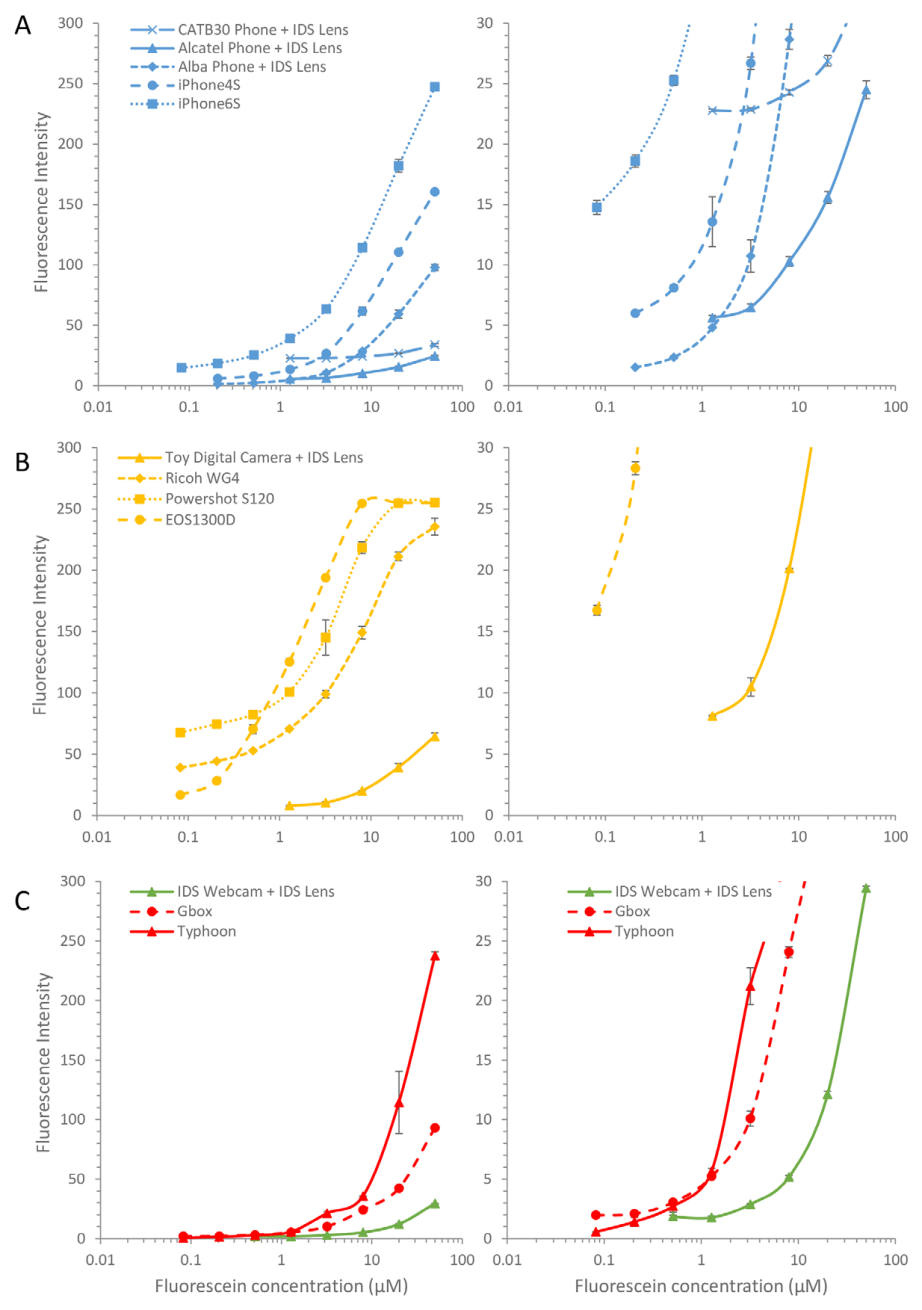
Quantitation of fluorescent microfluidic readout with a range of digital cameras and phones. The signal intensity for the images in Figure 4 were determined and mean fluorescence intensity for 10 individual capillaries plotted against concentration. **A**) Comparison of feature phones vs smartphones; where indicated a macro lens was used to be able to resolve individual capillaries. **B**) Comparison of consumer digital cameras. **C**) Comparison of industrial camera vs two laboratory fluorescent scanners. Error bars indicate the standard deviation of 10 capillaries. Lower intensity values are plotted (Right) with smaller y-axis range to permit comparison of detection of limiting concentrations of fluorophore.

The least effective fluorescence detection within the group of phone cameras was with the two feature phones (Alcatel and CAT B30), which although equipping with the macro lens for closer focussing permitted individual capillaries to be clearly resolved, very limited difference in intensity was measured across a wide range of fluorescein dye ( Figure 5A). In contrast, the budget smartphone (Alba Phone) gave adequate quantitation of fluorescence with the macro lens. This difference could be influenced not only by the sensor and lens, but also possibly by the image acquisition software. The budget smartphone has a far bigger screen and far better processing power onboard than the two feature phones, that may permit improved imaging of fluorescence within microfluidic devices. However, the higher performance smartphones (iPhone 4S and iPhone 6S) were clearly superior at quantitation of fluorescent microfluidic devices, showing clear quantitation below 0.2 µM and 0.5 µM respectively. The digital cameras were all far better at quantitation of fluorescence than colorimetric dye, with the two compact digital cameras performing similarly to the DSLR and all capable of measuring 0.2 µM fluorescein ( Figure 5B). However, the higher concentrations of fluorescein were saturated for two of the digital cameras, suggesting that careful utilisation of what are typically 8-bit intensities (i.e. 256 shades) is needed to maximise assay dynamic range. Even the cheapest toy camera was capable of adequate quantitation, although at far higher concentrations – with the first steep rise in intensity visible from 2 µM up to 8 µM fluorescein – and requiring the macro lens for close focussing. Again, the industrial machine vision camera gave less sensitive detection of fluorescence than many of the consumer digital cameras and phone cameras, and the macro lens was essential for close focussing to resolve individual capillaries. The two laboratory scanners, designed for scanning fluorescence, proved no better at quantitation of fluorescence within microfluidic devices than the consumer digital cameras ( Figure 5C).

### Antiviral antibody levels can be measured using a wide range of smartphones, cameras or laboratory imaging systems

**Figure 6.**
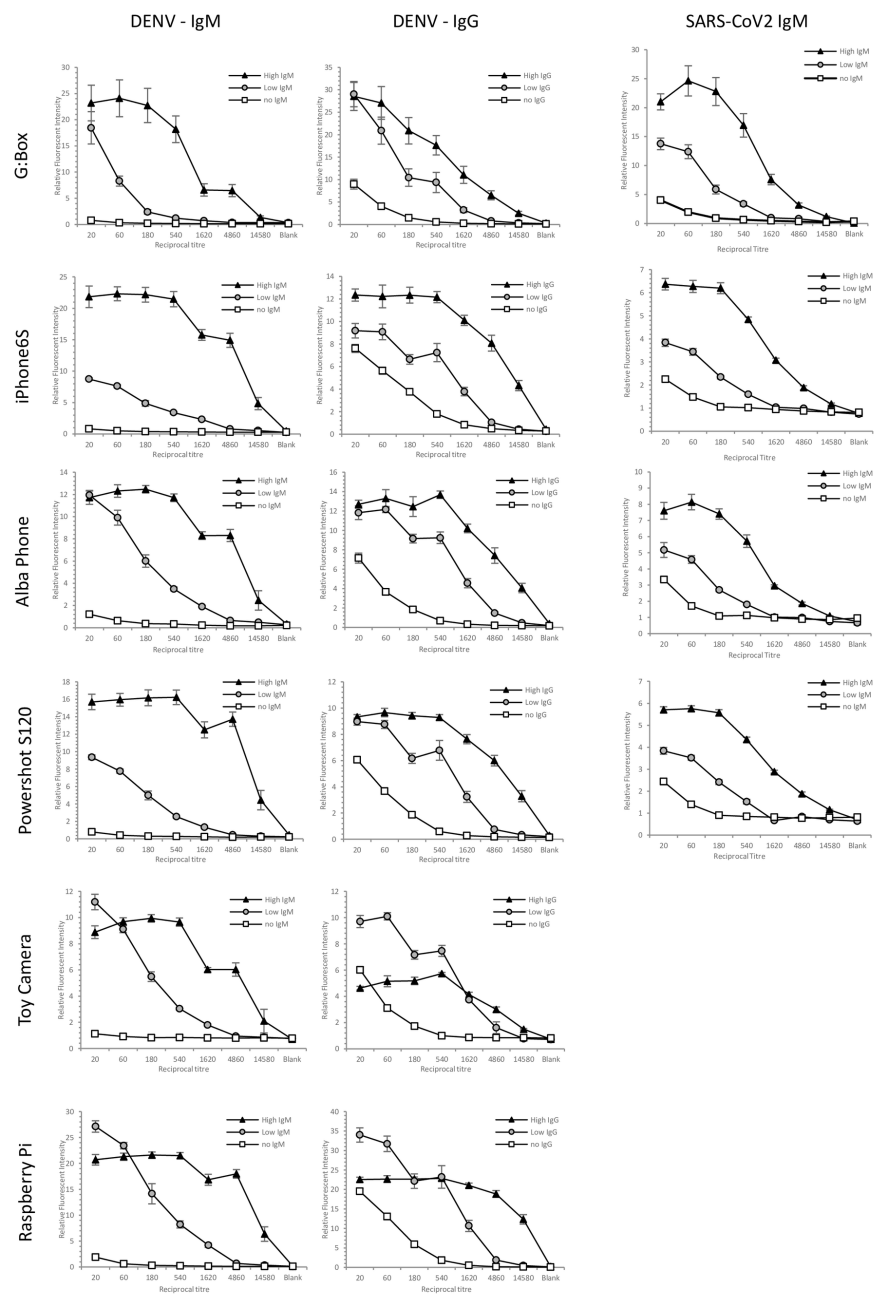
Comparison of a wide range of digital cameras for recording microfluidic immunoassays to measure antiviral antibody. Three simulated patient samples were made by spiking two different levels of recombinant antiviral antibodies into human serum, plus a negative control. Simulated IgM anti-dengue (Left), recombinant IgG anti-dengue (Middle) and recombinant IgM anti-SARS-CoV2 samples were serially diluted and 7 dilutions plus one blank (no serum) tested in microcapillary film (MCF) test strips coated with the respective viral protein, followed measuring human antibody level by enzyme fluorescence. The same set of 8 samples were imaged by the indicated cameras or scanner, and mean fluorescence intensity for 10 individual capillaries plotted; error bars indicated the standard deviation of 10 capillaries. These data are representative of three or more independent assays and camera comparisons.

Anti-viral antibody measurement assays were selected to illustrate fluorometric bioassays where microfluidic point-of-care and testing outside a lab could be valuable, especially in resource limited regions where use of low-cost consumer digital cameras is of greatest benefit. We wanted to demonstrate the quantitation of bioassays was largely unaffected by camera type, and we therefore made use of recombinant positive control antibodies spiked into negative control plasma to provide uniform assays. Indirect immunoassays with multiple patient sample dilution are typically used to quantify levels of antibody against viral antigens, for example for serosurveillance programs. Although we published proof-of-concept of indirect immunoassays using mouse antibodies, here we used recombinant humanised monoclonal antibodies spiked into human plasma. Microcapillary film strips internally coated with DENV2 E protein or SARS-CoV-2 S protein were used to measure reactivity at multiple dilutions of simulated plasma samples, and after assay was completed were imaged using the indicated cameras. With all cameras, all response curves showed the expected dilution-intensity relationship ( Figure 6), with both IgM assays only showing weak signal at the lower dilutions (1:20 and 1:60) vs the far higher signal that is maintained after multiple serial dilutions for the spiked positive control samples. For anti-DENV IgG the background was somewhat higher at these lower dilutions for control samples, as expected given the higher levels of IgG found in plasma, but the spiked positive control still showed far higher signals when more highly diluted. Both the stand-alone camera module and a laboratory scanner showed similar performance to the consumer digital cameras, with a Raspberry Pi 3 B + connected to the 8MP camera module v2, representing an open source and lower cost version of the IDS industrial machine vision camera.

Each set of dilutions represented 8 test strips, each with 10 replicate capillaries, hence each individual image captured 80 data points. For this comparison of cameras with immunoassays, the full set of test strips were imaged in one single image, but for the lowest resolution camera (the toy camera) it was not possible to distinguish clearly distinct capillaries (see Figure 1B for examples), yet clear differences in intensity were measured for the whole strip.

All the imaging systems gave similar response curves with exception of the toy camera, which showed reduced signal for the strong positive control in the IgG assay. Furthermore, the low-quality image with the toy camera made it hard to distinguish individual capillaries, so multiplex analysis would not be feasible with distinct bioassays in each of the 10 capillaries. Thus although the lowest performance (and cheapest) camera was still capable of quantifying overall fluorescent intensity and thus capably of quantifying microfluidic immunoassay results, the reduced analytical performance combined with an inability to capture as many test strips in a single photo makes it less suitable to quantitative clinical measurements. We conclude that a very wide range of digital imaging devices including smartphones, feature phones, consumer digital camera, and camera modules are all effective at recording clinically important immunoassays in microfluidic devices. Higher resolution digital imaging can offer higher density of data capture. All original image files are shared through the experimental dataset accompanying this paper.

### Critical parameters for successful digital imaging of microfluidic devices

We suggest the following framework for selecting a camera for digital imaging of microdevices and bioassays. Firstly, establish the clinical need and analytical requirements (i.e. measurement range, limit of detection) and thereby define the number and size of devices that need to be recorded. Alongside this, it is important to understand any regulatory constraints such as any need to have complete control over software or camera settings. Whilst separate from the technical requirements evaluated in this study, such regulatory or software requirements may rule-out use of consumer products. Once the number and size of devices are defined, both the overall image size needed (camera field of view) and the resolution required to quantify individual test areas can be established. As far as we could establish in this study, as long as the image acquired can resolve individual microdevice channels i.e. each analytical chamber can be clearly identified on the final image, the camera will be able to quantify signal. We do suggest real-world images are taken to establish image resolution however, as the observed resolution may not match manufacturers claimed performance. The working distance from camera to devices may also influence setup. Finally, the dynamic range of quantitation of signal, and analytical sensitivity required must both be considered. It may be necessary to modify camera settings to expand dynamic range or to match the required analytical sensitivity.

## Conclusion

We found that a wide range of digital cameras – including the lowest cost consumer products – were capable of recording and quantifying fluorescence and colorimetric signal in 200 µm diameter channels within microfluidic devices. Nevertheless, there are clear benefits of higher performance optoelectronics modules, both in terms of sensitivity and quantitation of lower concentrations of target signal, and in number of microfluidic channels that can be simultaneously captured. Likewise, camera optics had some influence on detection, but even addition of a very simple macro lens was sufficient to allow low resolution digital cameras to capture and quantify microfluidic device signal. With the higher performance cameras, a higher resolution image made it easier to resolve individual microfluidic channels, and allowed simultaneous capture of larger numbers of microdevices. Finally, we found that that most digital cameras including budget smartphones are very capable of capturing and quantifying fluorescent microfluidic immunoassays to measure antibody responses to two viral infections of global health significance: dengue fever and COVID-19. Overall, this study highlights the range of performance requirements for digital capture of miniature diagnostic tests, and provides a framework to develop a clear specification combining the clinical diagnostic application with the device type, to permit use of the lowest cost digital camera for result capture, analysis and quantitation.

## Data availability

The data referenced by this article are under copyright with the following copyright statement: Copyright: ï¿½ 2021 Jégouic SM et al.

Data associated with the article are available under the terms of the Creative Commons Attribution Licence, which permits unrestricted use, distribution, and reproduction in any medium, provided the original data is properly cited. 

### Underlying data

Figshare: Dataset associated with the article "Affordable mobile microfluidic diagnostics: minimum requirements for smartphones and digital imaging for colorimetric and fluorometric viral antibody detection". https://doi.org/10.6084/m9.figshare.13103414 ^26^.

This project contains the following underlying data:

- Images and list of imaging systems and conditions.

Data are available under the terms of the Creative Commons Attribution 4.0 International license (CC-BY 4.0).
