# Supplementary material for: Affordable mobile microfluidic diagnostics: minimum requirements for smartphones and digital imaging for colorimetric and fluorometric anti-dengue and anti-SARS-CoV-2 antibody detection
Source: Wellcome Open Res. 2021 Mar 11;6:57. [Version 1] doi: 10.12688/wellcomeopenres.16628.1 (PMC9614285; doi:10.12688/wellcomeopenres.16628.1)
Supplement: Peer review history

# Peer review: Reviewer response for version 1

**DOI:** 10.21956/wellcomeopenres.18332.r52711

**Published:** 2022-11-22

**Authors:** Zulfiqur Ali, Simon Bateson

**Affiliations:** 1 School of Health & Life Sciences, Healthcare Innovation Centre, University of Teesside, Middlesbrough, UK

This paper compares a wide range of cameras for use in miniaturised colorimetric and fluorometric bioassay. An interesting finding was that a wide variety of optical detector options are available for bioassay and what is required is both the overall image size that is needed and the individual test areas to respectively specify the camera field of view and the resolution. A key point was that if the image resolution was sufficient to identify individual fluidic channels then all cameras were able to quantify a similar range of concentrations of colorimetric and fluorometric dyes. Moreover, even the cheapest and lowest resolution cameras were able to record and quantify immunoassays.

 There was no mention in the paper about the need to exclude light. It is assumed that an assay would require either some processing within software or some form of phone mounting to exclude light. It was also interesting to note that the expensive industrial camera did not perform particularly well. Generally, these types of cameras are designed for fast accurate rendition in a well-controlled lighting environment. In contrast, phone cameras are generally designed to give a reasonable picture over the widest possible range of lighting using auto-exposure. There is a potential need for a reference within the picture, noise will vary with the signal so that a high absorbance or a low fluorescence will give a noisy signal.

Is the work clearly and accurately presented and does it cite the current literature?

Yes

If applicable, is the statistical analysis and its interpretation appropriate?

Yes

Are all the source data underlying the results available to ensure full reproducibility?

Yes

Is the study design appropriate and is the work technically sound?

Yes

Are the conclusions drawn adequately supported by the results?

Yes

Are sufficient details of methods and analysis provided to allow replication by others?

Yes

Reviewer Expertise:

Microfluidics and instrumentation.

We confirm that we have read this submission and believe that we have an appropriate level of expertise to confirm that it is of an acceptable scientific standard.

# Peer review: Reviewer response for version 1

**DOI:** 10.21956/wellcomeopenres.18332.r52712

**Published:** 2022-10-27

**Authors:** Anna N. Berlina

**Affiliations:** 1 Bach Institute of Biochemistry, Research Center of Biotechnology, Russian Academy of Sciences, Moscow, Russian Federation

The authors presented interesting experimental data when comparing different cameras used to capture images of microfluidic channels. This is important since, in experimental articles, one method of data processing is usually used and it is not very clear whether the camera resolution was enough and whether the settings were sufficient for sensitive determination of the analyte. Yes, this has been tested on model systems, but in this case it is even more clear how to reproduce it. There are some minor comments below.

1. Introduction, “smartphones ^1^, including pH in clinical samples” – these are two different directions? This part of sentence needs to be revised.
2. Introduction, the term “CMOS ” is not explained.
3. How the LOD was determined? It should be added to the Methods section.

Is the work clearly and accurately presented and does it cite the current literature?

Yes

If applicable, is the statistical analysis and its interpretation appropriate?

Yes

Are all the source data underlying the results available to ensure full reproducibility?

Yes

Is the study design appropriate and is the work technically sound?

Yes

Are the conclusions drawn adequately supported by the results?

Yes

Are sufficient details of methods and analysis provided to allow replication by others?

Yes

Reviewer Expertise:

Immunobiochemistry

I confirm that I have read this submission and believe that I have an appropriate level of expertise to confirm that it is of an acceptable scientific standard.

# Peer review: Reviewer response for version 1

**DOI:** 10.21956/wellcomeopenres.18332.r49530

**Published:** 2022-04-11

**Authors:** Dianping Tang

**Affiliations:** 1 Key Laboratory of Analytical Science for Food Safety and Biology (MOE & Fujian Province), Department of Chemistry, Fuzhou University, Fuzhou, China

This manuscript deals with an interesting work on affordable mobile microfluidic diagnostics. A comparative study was performed for the colorimetric and fluorometric detection of anti-dengue and anti-SARS-CoV-2 antibody by using smartphones and digital imaging for capturing and measuring the results of microfluidic bioassays. A set of 200µm diameter microchannels was filled with a range of concentrations of dyes in colorimetric and fluorometric enzymatic immunoassays. This work can be considered for indexing in this journal with appreciative revision.

**Specific comments:**

1. The use of digital cameras in consumer devices, such as smartphones, to record the results of miniaturised bioassays, offers many potential advantages in clinical diagnostics. Smartphones can digitally record, interpret and quantify many measurements that have previously relied on a laboratory instrument to analyse, or needed a human operator to interpret lines or colour changes by eye and then manually record results. Please provide the corresponding works for these descriptions (e.g. ^1^^,^^2^^, ^^3^ ).
2. Fluorescent readouts can offer higher analytical sensitivity than colorimetric detection, although the sensitivity of fluorescent detection depends significantly on excitation intensity and wavelength, quality of emission filters, in addition to the optical detector used. Please provide the corresponding works.
3. The mechanism for the colorimetric and fluorometric detection of anti-dengue and anti-SARS-CoV-2 antibody by using smartphones and digital imaging should be further discussed in the main text.
4. Please carefully check all references, and irrelative references should be removed throughout the text. Meanwhile, old literature should be updated, especially before 10 years.

Is the work clearly and accurately presented and does it cite the current literature?

Partly

If applicable, is the statistical analysis and its interpretation appropriate?

Yes

Are all the source data underlying the results available to ensure full reproducibility?

Yes

Is the study design appropriate and is the work technically sound?

Yes

Are the conclusions drawn adequately supported by the results?

Yes

Are sufficient details of methods and analysis provided to allow replication by others?

Yes

Reviewer Expertise:

Analytical Chemistry

I confirm that I have read this submission and believe that I have an appropriate level of expertise to confirm that it is of an acceptable scientific standard.

## References

[1] : Ti3C2 MXene quantum dot-encapsulated liposomes for photothermal immunoassays using a portable near-infrared imaging camera on a smartphone. *Nanoscale* .2019;11(33) : 10.1039/c9nr05797h 15659-15667 10.1039/c9nr05797h 31411624

[2] : A new visual immunoassay for prostate-specific antigen using near-infrared excited CuxS nanocrystals and imaging on a smartphone. *Analyst* .2019;144(12) : 10.1039/c9an00724e 3716-3720 10.1039/c9an00724e 31134993

[3] : Self-Referenced Smartphone Imaging for Visual Screening of H2 S Using Cu O-Polypyrrole Conductive Aerogel Doped with Graphene Oxide Framework. *Analytical Chemistry* .2018;90(16) : 10.1021/acs.analchem.8b03011 9691-9694 10.1021/acs.analchem.8b03011 30051716
